# Clinical value of mammography in diagnosis and identification of breast mass

**DOI:** 10.12669/pjms.324.9384

**Published:** 2016

**Authors:** Hongjun Li, Shanhua Zhang, Qingyuan Wang, Rongguang Zhu

**Affiliations:** 1 Hongjun Li, Department of Radiology, Binzhou People’s Hospital, Binzhou, Shandong, 256610, China; 2 Shanhua Zhang, Department of Radiology, Binzhou People’s Hospital, Binzhou, Shandong, 256610, China; 3 Qingyuan Wang, Department of Science and Education, Binzhou People’s Hospital, Binzhou, Shandong, 256610, China; 4 Rongguang Zhu, Department of Radiology, Binzhou People’s Hospital, Binzhou, Shandong, 256610, China

**Keywords:** Mammography, Breast cancer, Breast Imaging-Reporting and Data System-Mammography, Pathological diagnosis

## Abstract

**Objective:**

To study the effect and clinical value of mammography in the diagnosis of breast lump so as to improve the diagnosis level of breast cancer.

**Methods:**

A retrospective analysis was carried out on clinical data of 110 patients with mammary lump confirmed by pathology to study the compliance of mammography diagnosis and Pathology diagnosis in breast lump, and the detection of microcalcifications, phyllode, and observe the image performance of mammography. Taking infitrating ductal carcinoma (IDC) as an example, the correlation of image performance and clinical pathological features of different types was studied so as to predict if mammography performance was effective in the treatment and prognosis in breast cancer.

**Results:**

Taking Breast Imaging Reporting and Data System (BI-RADS) grade 4A as the critical point, the sensitivity, specificity and accuracy of mammography was 90.80% (109/120), 84.60% (126/149) and 87.40% (235/269); taking BI-RADS grade 4B as the critical point, the sensitivity, specificity and accuracy of mammography was 85.00% (102/120), 93.30% (139/149) and 89.60% (241/269); the correlation analysis suggested that, there was some kind of correlation between the mammography performance and clinical features of breast cancer.

**Conclusion:**

Mammography is worth being promoted in clinic for its significant clinical value in diagnosing and identifying breast lump.

## INTRODUCTION

Breast cancer is a type of malignant epithelial tumor with obvious local invasion capability and distant metastasis tendency. Because of breast cancer stem cells’ multi-lineage potential and the variance of individual microenvironment, the morphology performance of breast cancer is diversified, and some histological types have distinctive clinical features and prognosis significance.^1^ Breast cancer has become a disease influencing women’s health. Its incidence has great regional differences, usually developed areas with high incidence.^2^ During the last 20 years, the incidence of breast cancer has been on the rise, and age of onset tends to be young, but overall survival and disease-free survival tend to be longer.^3^ It is reported that it takes about 2 to 3 years from the beginning and when the lump of 1cm can be felt by physical examination.^4^ While during this period, most breast cancer will experience a process from localized in-situ to localized invasive to invasive growth, therefore, early detection, early diagnosis, and early treatment of breast cancer is of great significance, which can not only increase recovery rate, but also extend survival period, besides, it is important for female patients in maintaining good body feature, raising confidence and improving living quality.^5^

Currently, image examination is an important method in early diagnosis of breast cancer and the prognosis of patients, including ultrasonography, Magnetic Resonance Imaging (MRI) and mammography examination, etc.^6^,^7^ There are several common methods in the diagnosis of breast cancer, which is of both advantages and limitations. Two-dimensional ultrasonography is largely sensitive to individual techniques of operators, thus its susceptibility and specificity remains to be improved;^8^ MRI has good susceptibility and specificity, however, complicated examining techniques, long-time taking, poor patients’ compliance and it is insensitive to calcifications. Mammography is a widely used method in breast disease screening which has simple operation, little trauma, low cost and wide application, especially for the display of breast lumps’ shape and boundary, and diagnosis of sand-like calcification in lesions, and it has high accuracy.^9^ To evaluate the clinical value of mammography on breast lump, this study enrolled 238 patients pathologically confirmed with breast lumps, summarized the mammography diagnosis results of all patients and compared them with pathological results. Moreover, we discussed over the sensitivity, specificity and accuracy of mammography to breast lump when BI-RADS grade 4A and 4B were taken as the critical point.

## METHODS

A total of 238 females with breast lumps (269 lesions) who underwent pathological examination, preoperative ultrasonography and mammography in Binzhou People’s Hospital, Shandong, China from Aug, 2011 to Dec, 2013 were enrolled, with age ranging from 16~78 years (average 47.1±12.2 years). All breast lesions were graded by two experienced ultrasound doctors and radiologists according to grading criteria of Breast Imaging Reporting and Data System (BI-RADS). Patients who received preoperative anti-cancer therapy such as radiotherapy, chemotherapy, endocrine therapy, etc were excluded.

### Examination equipment and method

Mammography examination adopted Finland PLANMED digital mammographic apparatus, Kodak Diretview CR850 and KODAK DryviewCR8150 laser camera, with conventional photography position of craniocaudal (CC) view and medial-lateral oblique (MLO) view. If necessary, point photography or ampliphotography with automatic exposure were used. According to breast imaging-reporting and Data System-Mammography (BI-RADS-Mammography) category criteria of American College of Radiology (ACR), one experienced physician of image department read the image and issued diagnosis report.

### Grading criteria of BI-RADS^10^

Grade 0: further evaluation by imaging examination was needed; grade 2: negative and no abnormality; grade 2: sign of benign; grade 3: be benign lesion probably (possibility of malignant lesion < 2%) and short-term follow up was suggested; grade 4 (4A: low malignancy; 4B: medium malignancy, 4C: high malignancy): being suspected as malignant lesion (risks of malignancy: 3% ~ 94%), suggested to undergo biopsy; grade 5: be malignant lesion probably (risks of malignancy ≥ 95%); grade 6: be proven as malignant lesion by histopathological biopsy.

### Screening criteria for breast cancer with mammography^11^

major signs for breast cancer in mammography included lump, micro-calcification, local compact infiltration, burr-like boundary of lump or infiltration area and secondary signs included thickened skin, nipple retraction, more thickened blood vessels, comet tail sign and peritumoral edema ring. Once one major signs and two secondary signs were observed, then breast cancer could be confirmed. When X ray films suggested typical malignant calcification but no other malignant sings, it could be diagnosed as breast cancer.

### Statistical method

SPSS 19.0 statistical software package was used for statistical processing. Taking pathological examination results as diagnostic criterion, the diagnostic accuracy of mammography examination on breast cancer was evaluated and comparison was made. The enumeration data was expressed by percent (%) with chi-square test. *p* < 0.05 indicates the difference was statistically significant. SPSS19.0 was used for statistical analysis. Matched fourfold table data were examined by chi-square test and KAPPA consistency test. Difference was considered to be statistically significant if *p* < 0.05. The consistency was at a medium or high level when the K value was between 0.4 and 0.75; K ≥ 0.75 means high consistency; K ≤ 0.4 means poor consistency.

## RESULTS

### Mammography imaging characteristics of breast cancer in different pathological types

**Fig.1:**
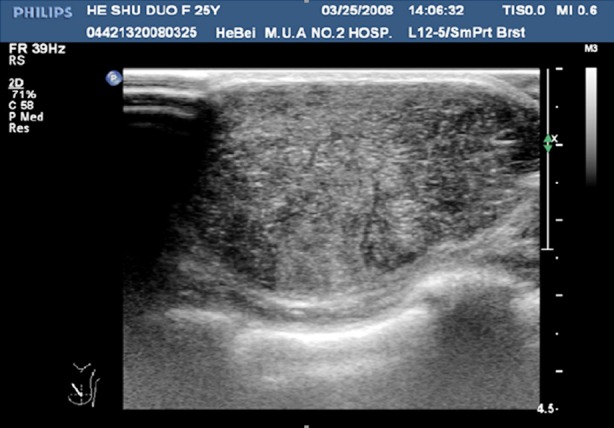
Ultrasonography of benign fibroadenoma.

**Fig.2:**
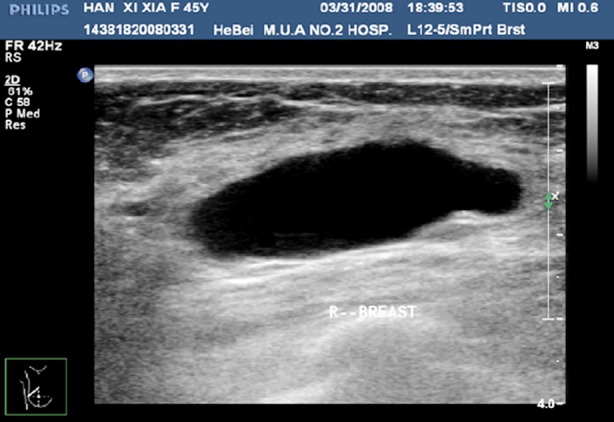
Ultrasonography of benign cyst.

**Fig.3:**
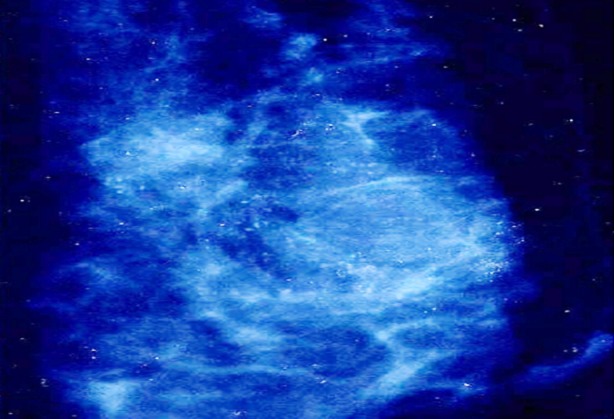
Mammogram shows clustered microcalcifications.

**Fig.4:**
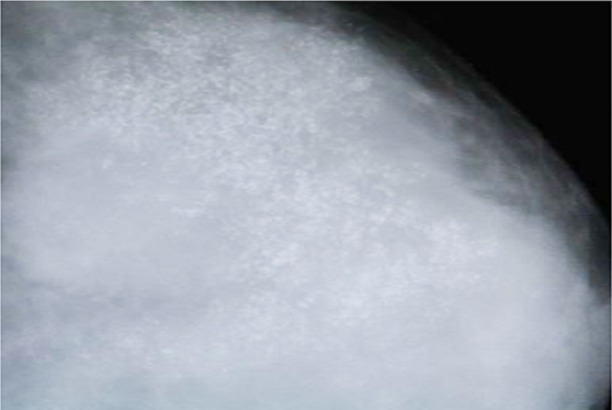
Mammogram shows granular microcalcifications in the malignant lesion.

**Fig.5:**
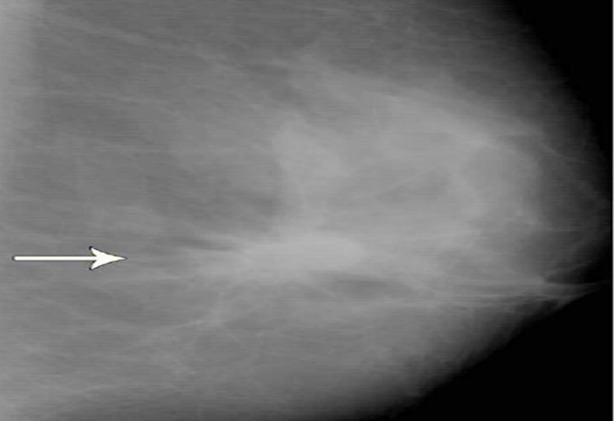
Mammographic image of two rounded opacities with indistinct borders.

**Fig.6:**
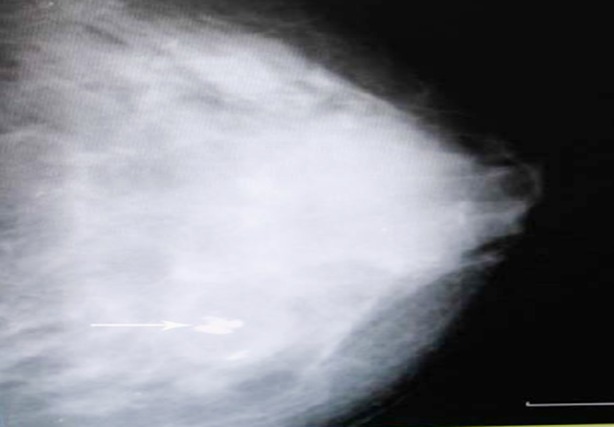
Mammogram shows coarse calcifications in the benign lesion.

One hundred forty nine cases were diagnosed as benign lesion, including 75 cases of adenoma, 48 cases of adenoma fibrosum (Fig.1), 14 cases of intraductal papilloma, three cases of mammitis, two cases of lipomyoma, two cases of phyllodes tumors, two cases of cyst (Fig.2), one case of granulomatous inflammation, 1 case of hyperplasia of mammary glands and 1 case of abscess. Of 120 cases that were diagnosed as malignant lesions, 86 cases were invasive ductal carcinoma, 17 cases were invasive lobular carcinoma, 4 cases were intraductal carcinoma, 3 cases were papillocarcinoma, 2 cases were low-malignant phyllodes tumor, 1 case was paget disease accompanied with intraductal carcinoma and 7 cases were invasive ductal carcinoma accompanied with lobular carcinoma. Mammography imaging characteristics differed among breast cancer in different pathological types; the specific performance included irregular boundary of lump, microcalcification, local compact infiltration, structural distortion, nipple retraction, thickening or retraction of local skin (Fig. 3 and 4), or clear breast anatomical structure, regular form, even density and thick calcification (Fig. 5 and 6). Table-I.

**Table-I:** Mammography imaging characteristics of 269 cases of breast lumps.

Pathological type	No.	Lump	Micro-calcification	Thick cal cification	Local compact infiltration	Thickened skin	Nipple retraction	Vascular anomaly
Invasive ductal carcinoma	86	50	28	12	26	6	12	15
Invasive lobular carcinoma	17	3	3		6			3
Adenoma fibrosum	48	27	1	8			2	3
Intraductal papilloma	14	3						
Lipomyoma	2	1						
Phyllodes tumor	2	2				1		1
Adenosis	75	27		7				
Cyst	2	2						
Inflammatory granuloma	1	1			1			
Mastitis	3	1						
Hyperplasia of mammary glands	1	1						
Abscess	1				1			
Intraductal carcinoma	4	4			2	2		
Papillocarcinoma	3	3			1			2
Low-malignant phyllodes tumor	2	1			1			
Paget disease accompanied with intraductal carcinoma	1	1		1				
Invasive ductal carcinoma accompanied by lobular carcinoma	7	5		4	2	2		

### Accuracy, sensitivity and specificity of mammography under different positive standard

If BI-RADS grade 4A or higher was taken as the positive standard, then the accuracy, sensitivity and specificity of mammography were 87.40% (235/269), 90.80% (109/120) and 84.60% (126/149); if BI-RADS grade 4B or higher was taken as the positive standard, then the accuracy, sensitivity and specificity of mammography were 89.60%(241/269), 85%(102/120) and 93.30% (139/149) (Table-II).

**Table-II:** Comparison of BI-RADS grading results and pathological results.

Pathological results	BI-RADS grade							Total

	1	2	3	4			5	

				4A	4B	4C		
Benign	3	66	57	13	5	3	2	149
Malignant	0	2	9	7	13	20	69	120

Total	3	68	66	20	18	23	71	269

### Analysis of related evaluation indexes

If BI-RADS grade 4A or higher was taken as the positive standard, the accuracy, sensitivity and specificity of mammography in diagnosing breast cancer were 87.40%, 90.80% and 84.60%; if BI-RADS grade 4B or higher was taken as the positive standard, then the accuracy, sensitivity and specificity of mammography were 89.60%, 85% and 93.30%. The above data were processed by KAPPA consistency test and the results were K = 0.157 (P = 0.055), K = 0.247 (P = 0.007) and K = 71.3% (P = 0.000). It indicated that, the sensitivity and specificity of mammography in diagnosing breast lump were poorly consistent with pathological results when BI-RADS grade 4B or higher was taken as the positive standard, but the accuracy was highly consistent. But compared to grade 4B, the sensitivity of mammography when grade 4A was taken as the standard had significant improvement, lowering the misdiagnosis rate. When BI-RADS grade 4A or higher was taken as the positive standard, the diagnostic index of mammography was 175.4%, higher than 170 %; and when grade 4B or higher was taken as the positive standard, then the diagnostic index of mammography was 178.3%, higher than 170%. Though the former percentage was lower than the latter one, grade 4A could be regarded as a necessary supplement when BI-RADS 4B or higher was taken as the positive standard, which might help lowering misdiagnosis rate.

## DISCUSSION

In clinic, breast cancer is a commonly seen malignant carcinoma in female and its incidence becomes increasingly higher, which is associated to the changes of dietary mode and living style.^12^ Early examination is the key for improving the survival rate of patients with breast cancer. In the early stage, breast lump is located in breast and has not been adhered to skin; hence it can be removed by surgery.^13^,^14^ Due to the small size of breast cancer, lack of specific clinical signs, misdiagnosis occurs frequently. Therefore, we rely mainly on auxiliary clinical examination by apparatus to check and find out early breast cancer before it was too late. And presently mammography is one of the main imaging examination means for breast cancer’s inspection.^15^,^16^ Digital mammography has been widely appreciated for its advantage of easy operation, less pain for patients, less X-ray exposure, clear image, and multiple processing ways for image, as well as its ability of providing accurate observation view for tumor edge, especially for micro speculation, deformed gland and fine calcification in lumps.^17^,^18^ With its increasingly prominent effect on reducing mortality rate of breast cancer, digital mammography has become a most common method in breast cancer screening, diagnosis, and follow-up period.

American College of Radiology formulated BI-RADS in 1992 and the version published in 2003 included BI-RAS-US, aiming to standardize the description of breast imaging, avoiding confusion, lower the technical difference between experienced doctors and inexperienced doctors and clarify definition of solid lesion.^19^

Mammography confirms malignant lesion based on the form and boundary of lump as well as the size, form, number and distribution of calcification. Diagnosis of breast cancer with mammography is established on the difference of density of lesion and surrounding tissue.^20^ In this study, among the lesions in grade 4A, there were 13 cases of benign lesions including two cases of adenoma, five cases of adenoma fibrosum, two cases of intraductal papilloma, one case of mastitis, one case of breast phyllodes tumor, one case of hyperplasia of mammary glands and one case of granulomatous inflammation and seven cases of malignant lesions. Surgical removal is the unique effective method for treating adenoma fibrosum. Intraductal papilloma is usually benign, with a malignancy rate of 6% ~ 8%. For lesions in grade 4B or higher, 8 cases were pathologically proven as benign lesions, including five cases of intraductal papilloma (2 cases of mild untypical hyperplasia and two cases of medium untypical hyperplasia), two cases of breast adenocarcinoma and one case of adenoma fibrosum. Intraductal papilloma is likely to have malignant transformation; but it is early to be discovered and usually can be removed timely.

### Limitations of the study

Even mammography examination had good early diagnosis effect on breast cancer, however, for internal breast area located at the edge of breast gland or breast tumor at deeper location near pectoralis, due to the limitation of technology itself, it is difficult to incorporate these lesions into film so one is unable-to-diagnose or cannot make diagnosis. For benign lesions, a few of them had blurred lump edges due to overlapped glands, or even with speculation, in this case, it is difficult to identify with malignant tumors only by mammography method; for young females with rich glands, some lesions can be overlapped by surrounding gland tissues which results in failure in diagnosis.

## CONCLUSION

Taking BI-RADS grade 4A or higher as the positive standard, the sensitivity of mammography in treating breast cancer was 90.80%; therefore, it is applicable for general investigation of breast cancer to lower the misdiagnosis rate of malignant tumor. Taking BI-RADS grade 4B or higher as the positive standard, the specificity of mammography in treating breast cancer was 93.30%, the diagnostic index was 178.3%, and the accuracy was relatively high; hence, grade 4B, 4C and 5 can be set as a surgical standard in clinic. BI-RADS grade 4A can be used for assisting positive diagnosis. Patients suffering from grade 4A breast cancer can be regarded as the key monitoring objects; regular ultrasonic review, surgical treatment and frozen pathology are required to timely discover malignant lesion and improve the survival rate of patients.
